# Understanding drugs in breast cancer through drug sensitivity screening

**DOI:** 10.1186/s40064-015-1406-8

**Published:** 2015-10-15

**Authors:** Katharina Uhr, Wendy J. C. Prager-van der Smissen, Anouk A. J. Heine, Bahar Ozturk, Marcel Smid, Hinrich W. H. Göhlmann, Agnes Jager, John A. Foekens, John W. M. Martens

**Affiliations:** Department of Medical Oncology, Erasmus MC Cancer Institute, Erasmus University Medical Center, Postbus 2040, ‘s-Gravendijkwal 230, 3000 CA Rotterdam, The Netherlands; Division of Janssen Pharmaceutica, Johnson & Johnson Pharmaceutical Research and Development, Turnhoutseweg 30, 2340 Beerse, Belgium; Department of Medical Oncology and Cancer Genomics Netherlands, Erasmus MC Cancer Institute, Erasmus University Medical Center, Postbus 2040, ‘s-Gravendijkwal 230, 3000 CA Rotterdam, The Netherlands

**Keywords:** Drugs, Screening, Cell line, Subtype, Pathway, Breast cancer

## Abstract

With substantial numbers of breast tumors showing or acquiring treatment resistance, it is of utmost importance to develop new agents for the treatment of the disease, to know their effectiveness against breast cancer and to understand their relationships with other drugs to best assign the right drug to the right patient. To achieve this goal drug screenings on breast cancer cell lines are a promising approach. In this study a large-scale drug screening of 37 compounds was performed on a panel of 42 breast cancer cell lines representing the main breast cancer subtypes. Clustering, correlation and pathway analyses were used for data analysis. We found that compounds with a related mechanism of action had correlated IC_50_ values and thus grouped together when the cell lines were hierarchically clustered based on IC_50_ values. In total we found six clusters of drugs of which five consisted of drugs with related mode of action and one cluster with two drugs not previously connected. In total, 25 correlated and four anti-correlated drug sensitivities were revealed of which only one drug, Sirolimus, showed significantly lower IC_50_ values in the luminal/ERBB2 breast cancer subtype. We found expected interactions but also discovered new relationships between drugs which might have implications for cancer treatment regimens.

**Electronic supplementary material:**

The online version of this article (doi:10.1186/s40064-015-1406-8) contains supplementary material, which is available to authorized users.

## Background

Life expectancy and survival of breast cancer patients have increased significantly over the last decades, due to—amongst other factors—an increasing number of effective drug therapies (Berry et al. 2005; Lichtenberg 2009, 2011). Drug resistance remains a major issue (Gonzalez-Angulo et al. 2007) and since the discovery that expression of the protein markers ER, PR and her-2/neu determines response to a given targeted therapy (Bast et al. 2001), the assessment of their expression in breast cancer has become an important first step in selecting a patient’s treatment (Bast et al. 2001). Subsequently, microarray studies have shown insight into molecular processes active in the tumor and linked those to diverse clinical outcomes (Sorlie et al. 2001; Van’t Veer et al. 2002; Wang et al. 2005) including therapy failure (Jansen et al. 2005). In the last couple of years large scale next generation sequencing efforts have made a big contribution to our understanding of breast cancer by delivering precise information on cancer driver mutations (Kangaspeska et al. 2012; Desmedt et al. 2012; Previati et al. 2013; Radovich et al. 2013; The Cancer Genome Atlas Network 2012). All these sources of information combined have helped to elucidate how breast cancer evolves, progresses and metastasizes and some of them have led to the development of diagnostic tests to characterize breast cancer better (Kittaneh et al. 2013). Nevertheless, there is still significant room for improvement in regard to available drug therapies, as many patients do not respond to current treatments or become resistant during the course of treatment (Gonzalez-Angulo et al. 2007). New agents are therefore needed to target breast cancer, and screenings of multiple compounds for their activity against the various breast cancer subtypes are a good starting point. As a first step to test new compounds breast cancer cell lines are a good model, because they are easy to maintain, represent different subtypes of breast cancer, and the response to drug treatment can be easily assessed. For these reasons, we studied the activity of a wide variety of cytotoxic and targeted drugs in a large panel of breast cancer cell lines. The drugs were chosen based on current clinical utility e.g. for discrete cancer subtypes, potential clinical utility such as promising compounds in pre-clinical testing, aiming at molecular targets, and—for comparison—current state of the art drugs for the therapy of breast cancer. We investigated which drugs showed similar activity in the panel of breast cancer cell lines and could therefore potentially substitute or complement each other in the clinic, and, in addition, we aimed to identify shared biology in cell lines that are affected by highly correlated drugs.

## Results

### Relationships between drugs: clustering and correlation analysis

**Fig. 1:**
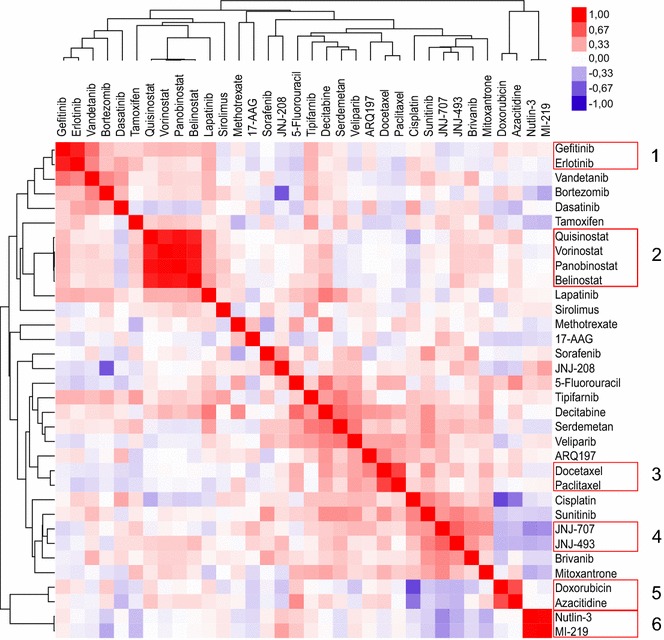
Pearson correlation plot of absolute drug IC_50_ values. The *red color* indicates a positive correlation between the IC_50_ values of two drugs, and *blue* a negative correlation. The *color intensity* illustrates the correlation coefficient as shown in the legend at *top right*. Drugs are clustered on the basis of similarity; distances in the tree indicate the degree of difference between drugs

**Fig. 2:**
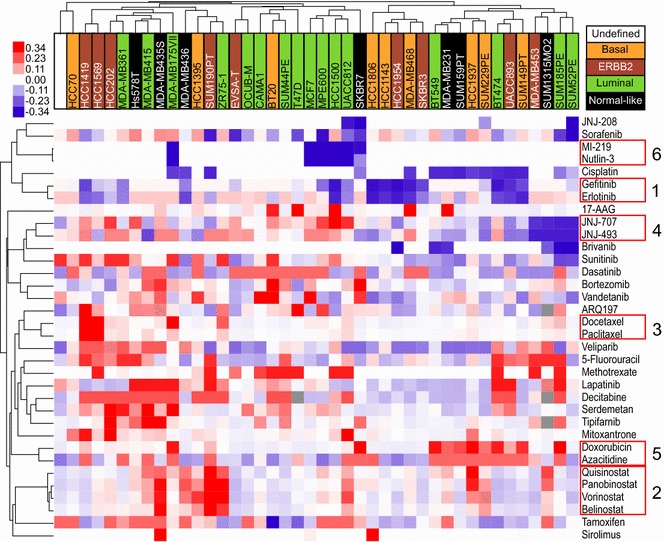
Similar drugs cluster together. Depicted is a hierarchical unsupervised clustering of the analyzable drugs and cell lines. *Blue color* indicates low IC_50_ values (i.e. cells are drug-sensitive), and *red color* high IC_50_ values (i.e. cells are drug-resistant). *Color intensity* illustrates the degree of drug sensitivity or resistance; outliers exceeding the legend boundaries are set to the maxima colors of the legend to ensure visibility of small differences instead of few outliers. Breast-cancer subtypes are color-coded on the basis of the intrinsic subtypes of breast cancer cell lines as previously described (Riaz et al. 2013). The respective legend can be found on the *top right*. Tree distance is representative for similarity of drugs or cell lines. Drugs with similar response profiles among the cell lines are highlighted by *red boxes*

To investigate the relationships between different drugs the IC_50_ values of all 7 cytotoxic drugs and 30 targeted agents, measured in the 42 breast cancer cell lines, were correlated (Fig. 1). Capecitabine, cMet 605 and Cyclophosphamide exhibited no differential IC_50_ values and were consequently omitted from the clustering and further analyses. To express the relationships among drugs and cell lines hierarchical clustering was performed (Fig. 2). Clustering and correlation performed fairly similarly and are therefore discussed together.

**Fig. 3:**
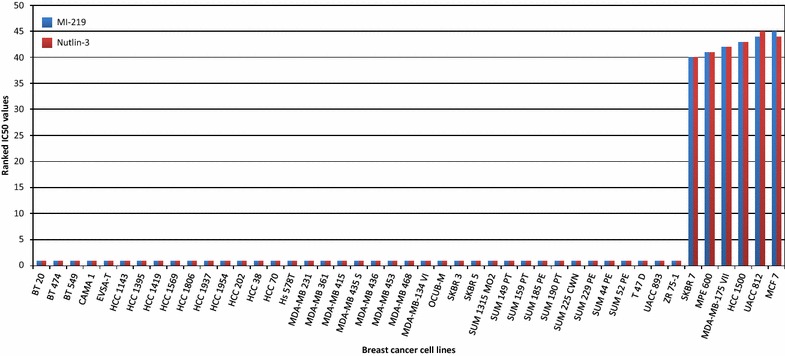
Nutlin-3 and MI-219 have similar drug sensitivity profiles among the cell lines (relative IC_50_ values). The relative IC_50_ is inverted, with high numbers indicating sensitivity in this case and not resistance. Few cell lines are sensitive to these drugs, while the majority is resistant

Strong correlation and expected co-clustering was observed between Gefitinib and Erlotinib (cluster 1; r = 0.88), between Quisinostat, Panobinostat, Vorinostat and Belinostat (cluster 2; r = 0.85–0.96), between Docetaxel and Paclitaxel (cluster 3; r = 0.73), between JNJ-707 and JNJ-493 (cluster 4; r = 0.62) and between MI-219 and Nutlin-3 (cluster 6; r = 0.98); all correlations are listed additionally in Table 1. To illustrate the close relationship between related drugs, the IC_50_ values of MI-219 and Nutlin-3, the two drugs with the highest correlation, were ranked and plotted for all cell lines (Fig. 3). Interestingly, Serdemetan, a drug which acts on cholesterol transport but also targets MDM2 (Jones et al. 2013)—a mechanism shared with Nutlin-3 and MI-219 (Shangary and Wang 2009)—showed no correlation with these two compounds.

**Table 1:** Correlated drugs

Drug 1	Drug 2	p-value	Pearson correlation coefficient
MI-219	Nutlin-3	1.77E−28	0.98
Panobinostat (Faridak^®^)	Vorinostat (Zolinza^®^)	2.14E−24	0.96
Panobinostat (Faridak^®^)	Quisinostat	1.66E−19	0.93
Belinostat	Vorinostat (Zolinza^®^)	2.05E−18	0.92
Belinostat	Panobinostat (Faridak^®^)	1.70E−16	0.91
Erlotinib (Tarceva^®^)	Gefitinib (Iressa^®^)	3.49E−14	0.88
Quisinostat	Vorinostat (Zolinza^®^)	1.05E−13	0.87
Belinostat	Quisinostat	8.08E−13	0.85
Paclitaxel (Taxol^®^, OnxalTM)	Docetaxel (Taxotere^®^)	4.61E−08	0.73
Azacitidine (Vidaza^®^)	Doxorubicin (Adriamycin^®^)	3.77E−07	0.7
JNJ-493	JNJ-707	1.39E−05	0.62
Decitabine (Dacogen^®^)	5-Fluorouracil	7.77E−05	0.58
Decitabine (Dacogen^®^)	Serdemetan	1.17E−04	0.56
Vandetanib (Zactima^®^)	Gefitinib (Iressa^®^)	1.52E−04	0.56
Serdemetan	Tipifarnib (Zarnestra^®^)	5.15E−04	0.52
Decitabine (Dacogen^®^)	Lapatinib	5.29E−04	0.52
Veliparib	Serdemetan	5.47E−04	0.51
JNJ-493	Sunitinib (Sutent^®^)	1.37E−03	0.48
Veliparib	Decitabine (Dacogen^®^)	1.63E−03	0.48
Vandetanib (Zactima^®^)	Erlotinib (Tarceva^®^)	1.78E−03	0.47
Bortezomib (Velcade^®^)	Vandetanib (Zactima^®^)	1.94E−03	0.47
ARQ197	Docetaxel (Taxotere^®^)	1.95E−03	0.47
Cisplatin	Sunitinib (Sutent^®^)	2.00E−03	0.47
JNJ-707	Brivanib	2.16E−03	0.46
Mitoxantrone (Novantrone^®^)	JNJ-707	2.98E−03	0.45
JNJ-707	Nutlin-3	2.87E−03	−0.45
Cisplatin	Azacitidine (Vidaza^®^)	2.16E−04	−0.55
JNJ-208	Bortezomib (Velcade^®^)	1.96E−06	−0.66
Cisplatin	Doxorubicin (Adriamycin^®^)	5.22E−08	−0.73

Correlation pairs were determined using IC_50_ values. Statistical thresholds for significance were defined as a p-value <0.01 and a Pearson correlation coefficient above 0.45 or below −0.45

Unanticipated but highly significant correlations were observed between particularly Doxorubicin and Azacitidine (cluster 5; r = 0.70), between Decitabine and 5-Fluorouracil (r = 0.58) and Serdemetan (r = 0.56); and between Serdemetan and Tipifarnib (r = 0.52). Additional weaker, but expected correlations were found for Vandetanib with Erlotinib and Gefitinib (r = 0.47, r = 0.56). Decitabine was correlated with Lapatinib (r = 0.52) and Veliparib with Serdemetan and Decitabine (r = 0.51, r = 0.48). Furthermore, we also detected a remote relation between various tyrosine kinase inhibitors like JNJ-493 with the multi-receptor tyrosine kinase inhibitor Sunitinib (Keyvanjah et al. 2012) (r = 0.48), JNJ-707 with FGFR- and VEGFR-inhibitor Brivanib (Huynh et al. 2008) (r = 0.46) and between Docetaxel and ARQ197 (r = 0.47). The DNA targeting drug Cisplatin (Becker et al. 2014) showed surprisingly a correlation with Sunitinib (r = 0.47); Bortezomib was correlated with Vandetanib (r = 0.47) and the type II topoisomerase inhibitor Mitoxantrone (Hajihassan and Rabbani-Chadegani 2009) was correlated with JNJ-707 (r = 0.45). In total, 25 pairs of positively correlated drugs were found.

Apart from positive correlations—and even more interesting—we also discovered significant negative correlations between certain drugs (Table 1). Particularly, Doxorubicin and the correlated drug Azacitidine had negative correlations with Cisplatin (r = −0.73, r = −0.55), the ERR1 targeting JNJ-208 with Bortezomib (r = −0.66), and the MDM2-targeting Nutlin-3 (Shangary and Wang 2009) with the FGFR inhibitor JNJ-707 (r = −0.45).

### Shared pathways between correlated drugs

**Fig. 4:**
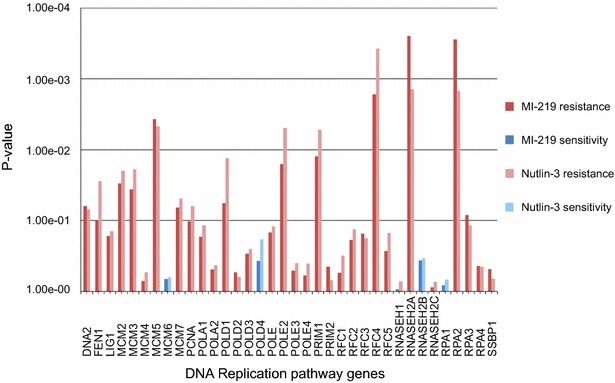
Differentially expressed genes of the DNA replication pathway for Nutlin-3 and MI-219. *Bar graphs* display the differentially expressed genes of this pathway between resistant and sensitive cell lines for Nutlin-3 and MI-219. *Red shades* indicate an association with resistance, *blue shades* indicate an association with sensitivity

To further understand the biology behind correlated drugs we used mRNA expression data of the untreated cell lines and the pathway information of the databases Biocarta and KEGG (Ogata et al. 1999) to characterize drug resistance in R (R_Core_Team 2013). We identified significant pathways for each of the evaluable drugs, but focused on the pathways which were shared among correlated drugs, i.e. for the 23 positively and 3 negatively correlated remaining drug–drug pairs. Furthermore, we performed a pathway analysis where cell lines were grouped per subtype to identify subtype-related pathways. Subtype-specific pathways were excluded from further study in the pathway-drug resistance analysis. At a significance level of p < 0.01, only one of all 26 correlation pairs had pathways in common. This pair, Nutlin-3 and MI-219, had, after correction for subtype-specific pathways, only the DNA replication pathway in common. The Nutlin-3- and MI-219-associated genes of this pathway are displayed in Fig. 4.

### Breast cancer subtype specific drugs

Earlier, several subtype-specific differences in drug sensitivity were observed (Heiser et al. 2012) and since breast cancer subtypes are biologically very different (Parker et al. 2009), we also explored whether drug response in our study was ER- or subtype-related. Only one drug, Sirolimus, exhibited a significantly different subtype-specific effectiveness. Normal-like and basal cell lines were more resistant to this drug compared to luminal and ERBB2-overexpressing cell lines with a change in sensitivity of two orders of magnitude (p = 0.005). The expression of ER by the latter two subtypes was not the sole explanation though, as none of the screened drugs was associated with ER status (p value >0.01).

## Discussion

### Drug response to one drug indicates the response to another

To understand drug resistance in breast cancer, we compared drug sensitivity of a large set of drugs within a large panel of breast cancer cell lines. It became evident that some drugs target breast cancer cell lines similarly and thus may have unanticipated overlapping mechanisms while others display opposing effects indicating that vulnerability to a given drug is protective for another unrelated treatment.

The results of the overall clustering (Fig. 2) show that every breast cancer cell line had a unique drug response profile, which might be true for patients as well. This—first—observation underlines the personal factor in drug sensitivity, which we need to understand upfront to provide optimal patient care.

The second, expected, conclusion is that drugs with identical targets such as MDM2-antagonists (MI-219 and Nutlin-3) (Shangary and Wang 2009), EGFR-inhibitors (Gefitinib and Erlotinib) (Cohen 2003), FGFR-inhibitors (JNJ-707 and JNJ-493), HDAC inhibitors (Quisinostat, Panobinostat, Vorinostat, Belinostat) (Lemoine and Younes 2010) and taxanes (Docetaxel and Paclitaxel) (Hagiwara and Sunada 2004), showed correlated sensitivities and clustered together explaining five of the six observed clusters.

More interesting was the third observation that unrelated drugs showed co-clustering, which is best exemplified by the sixth cluster (Figs. 1, 2), made up of the positively correlated intercalating agent Doxorubicin (Frederick et al. 1990) and the DNA-methyltransferase-targeting Azacitidine (Creusot et al. 1982). Interestingly and remarkably, Decitabine, a derivative of Azacitidine (Lyko and Brown 2005), which also targets a DNA-methyltransferase (Creusot et al. 1982), did not cluster with these two drugs. The reason for this might be that both Azacitidine and Doxorubicin have, next to their well-known properties, also the less known capability to interfere with RNA synthesis (Momparler et al. 1976; Christman 2002), while Decitabine can only act on DNA (Christman 2002). Next to this most notable finding we also observed a less strong correlation of Decitabine sensitivity with sensitivity to various unrelated drugs, i.e. the thymidylate synthetase inhibitor 5-Fluorouracil (Longley et al. 2003), the cholesterol transport inhibitor and MDM2-antagonist Serdemetan (Lehman et al. 2013; Jones et al. 2013), the EGF-receptor- and HER2-inhibitor Lapatinib (Huang and Rizzo 2012) and the PARP-inhibitor Veliparib (Glendenning and Tutt 2011). Some of these drugs additionally correlated with each other. Although several of these compounds target DNA synthesis and/or repair, there is no real common denominator between them. While these drugs could be targeted by the same drug efflux pumps, we could not find any among the drug-associated genes (pre-treatment gene expression) and suspect another, unknown mechanism. The same holds true for the associations of Serdemetan with Veliparib and the farnesyltransferase inhibitor Tipifarnib (Armand et al. 2007).

Additionally, we also observed a rather surprising correlation between Docetaxel, which disorganizes microtubules (Hagiwara and Sunada 2004), and the c-met kinase inhibiting agent ARQ197 (Scagliotti et al. 2013). However, supporting our findings, ARQ197 has also been linked to inhibition of microtubuli polymerization recently (Katayama et al. 2013).

Finally, sensitivity to Bortezomib, a proteasome inhibitor (Teicher et al. 1999), was also predictive of sensitivity to the multi-kinase inhibitor Vandetanib (Sathornsumetee and Rich 2006)—the combination of these two drugs is currently in clinical trial testing (Gramza et al. 2013). Thus, a protein or complex whose stability is proteasome-dependent, may affect sensitivity to this multi-kinase inhibitor. Furthermore, Cisplatin and JNJ-493 were somewhat correlated to Sunitinib sensitivity and JNJ-707 weakly correlated with response to Mitoxantrone, findings which remain to be understood.

The fourth finding was the absent or poor correlation of drugs acting on the same target, such as Serdemetan which was not correlated with the other two well-known and highly correlated MDM2-inhibitors Nutlin-3 and MI-219 (Shangary and Wang 2009). Furthermore, Serdemetan lacked a correlation with TP53 mutation status (data not shown) highlighting that this putative MDM2-inhibitor acts differently from the other MDM2-inhibitors. This unexpected observation can be explained by the additional characteristics of Serdemetan, inhibition of the cholesterol transport and the increased degradation of ABCA1 (Jones et al. 2013). Clearly these additional properties dominate over the MDM2-inhibiting role. Moreover, another study confirms that sensitivity to Serdemetan is independent of TP53-mutation status (Jones et al. 2013).

Furthermore, we noted that Lapatinib and Vandetanib, two EGFR-antagonists (Nelson and Dolder 2006; Sathornsumetee and Rich 2006) neither clustered immediately next to Gefitinib or Erlotinib nor next to each other and the correlation coefficient was also lower than expected for Vandetanib, while Lapatinib did not correlate with the other EGFR-antagonists at all. In both cases this is less surprising as both Vandetanib and Lapatinib act on additional targets, like HER2 for Lapatinib (Nelson and Dolder 2006), and the two proteins VEGFR2 and RET Kinase for Vandetanib (Sathornsumetee and Rich 2006). We tried to support this hypothesis by correlating the drug response with mRNA expression data of EGFR, HER2, FGFR, VEGFR2, and RET Kinase for all those drugs. However, none of the correlations was significant, which might be different if pre-treatment protein expression data is used, as the proteins are the direct drug targets.

Similarly, Brivanib showed only a weak correlation with one of the FGFR-inhibitors, JNJ-707, in our panel, which might be due to its additional target VEGFR (Huynh et al. 2008).

### Drug response to one drug indicates resistance to another

The fifth interesting observation was that for some drugs sensitivity predicted insensitivity to another drug. For instance—and most prominent—Cisplatin resistance correlated with Doxorubicin sensitivity. Cisplatin’s mode of action involves DNA and RNA interstrand linkage (Stordal and Davey 2007), while Doxorubicin blocks DNA unwinding (Fornari et al. 1994), this difference does however not explain the clearly opposing character in drug response. The clinical implication might, nevertheless, be, that a patient showing insensitivity to Doxorubicin upon treatment start, might be more likely to respond to a course of Cisplatin therapy (Perilongo et al. 2009). Another implication of this finding is that a mechanism responsible for resistance to Doxorubicin reveals a target that provides synthetic lethality to Cisplatin or vice versa.

Previously, Cisplatin resistance was found to correlate with Taxane sensitivity (Stordal et al. 2007), a finding, we could not confirm in the present study.

The FGFR inhibitor JNJ-707 had an inverse correlation with Nutlin-3 response. Therefore, we also investigated whether TP53 wild-type cell lines (Riaz et al. 2013), which are sensitive to Nutlin-3, have a different expression of FGFR genes in contrast to mutant cell lines, but found no significant difference (data not shown). While a true biological effect cannot be excluded, it has to be mentioned that only few cell lines were sensitive to Nutlin-3 and our observation might be due to the low numbers. Finally, the proteasome inhibitor Bortezomib is negatively correlated to JNJ-208. Thus, if this observation implies causality, a proteasome-dependent mechanism affects vulnerability to this drug.

### Biology of correlated drugs

Next, we wanted to uncover shared biology of drug sensitivity in correlated drugs by performing a pathway analysis. The first thing we discovered was that there were hardly any shared pathways after excluding pathways with a strong subtype-association. This was rather surprising as we did not find many subtype-associated drugs, but can be explained by the analysis type since for the pathway analysis we used only the cell lines around the minimum and maximum drug response, while for the subtype analysis all cell lines were included. Hence the smaller number of cell lines might have introduced a bias, but generally, it seems that the biology driving the subtypes in breast cancer largely obscures the possible drug-related pathways. The two correlated drugs which had the DNA replication pathway in common were Nutlin-3 and MI-219. Nutlin-3 has been previously shown to downregulate proteins involved in DNA replication (Kumamoto et al. 2008), a process likely influenced by MI-219, as well. The cell lines which were sensitive to these drugs had intrinsically low expression of most pathway-associated genes pre-treatment and the drug-related shutdown of the remaining expression might be contributing to lethality.

From the results of the subtype-specific pathways it became obvious that normal-like cell lines are very different from luminal ones. However, when we did a global test to evaluate whether a certain breast cancer subtype showed an overall increased sensitivity to the tested compounds we found no differences. Therefore, we could not confirm a general drug resistance of normal-like cells which would be expected due to their mesenchymal and stem cell like properties (Al-Hajj et al. 2003; Ponti et al. 2006; Sieuwerts et al. 2009).

### Subtype-specific drugs

Of all tested drugs in this screening only one drug, Sirolimus, was more active in the luminal and ERBB2-high subtypes, as was noted previously in a comparable study (Heiser et al. 2012). However, in contrast to this earlier study (Heiser et al. 2012), who discovered 23 subtype-related drugs, we did not find subtype-dependent sensitivity for the other eight drugs screened in both studies. This discrepancy is likely due to several differences in study design, e.g. drug incubation times, type of readout assay, the use of collagen-coated plates in our study to mimic cellular context better and differences in the assayed cell lines to name a few.

## Conclusion

Through our cell line screening with new and well-known drugs, we found a number of interesting interactions between drugs of which several were not noticed earlier. Those findings have great potential for an application in the clinic as they might present opportunities when tumors show already resistance upon start of the treatment.

Next to expected similar sensitivity profiles for related drugs such as Gefitinib and Erlotinib, we also found opposing sensitivity profiles such as Cisplatin with Doxorubicin and confirmed one subtype-related drug, Sirolimus, which has been identified earlier. Further validation on the discovered positive and negative correlations and the subtype-specific drug are needed e.g. in the form of an animal study. In that aspect it would be very interesting to investigate whether animals with e.g. a Cisplatin-resistant tumor benefit from Doxorubicin treatment.

To conclude, our study provides new leads in the search for effective treatments especially in the context of inherent drug resistance.

## Methods

### Cell lines and drug screening

Forty-five breast cancer cell lines with confirmed identity (Riaz et al. 2013) and known origin (Hollestelle et al. 2010a) were cultured and screened in RPMI 1640 medium (Life Technologies, Paisley, UK) containing 10 % FBS (Lonza, Walkersville, USA). Ninety-six well collagen I-coated plates (BD Biosciences, San Jose, USA) were used for drug screening. Each drug—cell line combination was assayed in triplicate. Cells were seeded at the density required to reach the end of the exponential growth phase at 120 h of culture. Drug incubation was started 24 h post-seeding and lasted 96 h. For each drug 12 different dilutions were tested starting from 1.00^−5^ to 3.00^−11^ M (final concentration), except for Bortezomib (2.00^−5^ to 6.00^−11^ M), Sirolimus and 17-AAG (both: 2.00^−6^ to 6.00^−12^ M). Drug diluent was used as negative control treatment. DMSO was used as drug solvent and diluent for all drugs except for Methotrexate (drug solvent: 1 M NaOH, drug diluent: 0.9 % NaCl solution) and Cyclophosphamide (drug solvent: PCR-grade water, drug diluent: 0.9 % NaCl solution).

Three cell lines, SUM225CWN, MDA-MB-134VI, and SKBR5, failed in our drug screening due to slow growth or half-suspension growth, which is incompatible with the SRB assay, resulting in 42 cell lines for analysis.

### Assessment of drug sensitivity and IC_50_ calculation

Cell line growth was determined by measuring the total protein amount per well using the Sulforhodamine B Assay (SRB) (Voigt 2005): After the medium was discarded the cells were incubated with 10 % TCA for 60 min at 4 °C for fixation. Then the cells were thoroughly washed 5× with distilled water, air-dried and incubated with 0.4 % SRB solution for 2 h for protein-staining. Additional washing steps followed using 1 % acetic acid (4×) and cells were again air-dried. TRIS (10 mM) was added to the cells to dissolve the SRB overnight. Absorbances were measured at 570 nm in an Ascent MultiSkan (Thermo Electron Corporation, Waltham, USA). If required, samples were further diluted with TRIS to ensure optimal measurements. IC_50_ values were calculated using the respective absorbance values and are listed, besides all IC_50_ profiles, in the supplemental Excel file (Additional file 1).

### Clustering of cell lines and drug–drug correlation analyses

Cell lines were clustered in a hierarchical fashion based on their IC_50_ values. For drug correlation analysis, IC_50_ values per compound of each cell line were correlated with each other in Excel 2007 (Microsoft, Redmond, USA) using Pearson correlation. Cell lines with missing data for several drugs were discarded from correlation and cluster analysis, as were drugs that did not show differential IC_50_ values. The programs Cluster 3.0 (Eisen Lab, Stanford University, Stanford, USA) and Java Treeview version 1.1.6r2 (Saldanha 2004) were used to generate heatmaps of the correlation coefficients and the cluster analysis (Figs. 1, 2). Cluster 3.0 settings were as follows: normalize, median—center and average linkage using uncentered correlation as similarity metric. Figure 3 was generated in Excel. To generate the figures Inkscape 0.91 (Free Software Foundation Inc., Boston, USA) was used, next to the aforementioned programs.

### Association of signaling pathways with drug response

For pathway analysis we analyzed gene expression levels of cell lines with a high IC_50_- versus cell lines with a low IC_50_-value per drug. Cell lines were grouped in the high or low group by individual evaluation per drug instead of pre-selecting a fixed IC_50_ value for all drugs. This method was chosen to test the drug response extremes rather than testing values with little difference, which are present in gradual IC_50_ distributions. For this distribution type we used the cell lines at the distribution extremes and removed the intermediate values to reduce noise. A few drugs showed an IC_50_ profile that precluded a sensible grouping; e.g. Paclitaxel had only two cell lines with a high IC_50_ value while the others had a very low IC_50_; too few cell lines in a group renders the pathway analysis useless. For this reason, we excluded JNJ-208, Sirolimus, Docetaxel and Paclitaxel from the pathway analysis. For all other drugs, we were able to use meaningful group sizes of at least five samples each (Additional file 1).

For all cell lines, previously published mRNA expression data of our laboratory (Riaz et al. 2013) was used for pathway analysis (NCBI’s Gene Expression Omnibus database, entry GSE41313). Pathway analysis was performed using the Global Test package (Goeman et al. 2004) in R (R_Core_Team 2013) with information of the databases BioCarta LLC (San Diego, USA) and KEGG (Ogata et al. 1999). This R package was also used to generate Fig. 4, next to Excel 2007 and Inkscape 0.91. Furthermore, we also tested the identified pathways for a stronger association with breast cancer subtypes and disregarded those subtype-associated pathways (Additional file 2). The pathways significantly associated with a drug, including the subtype-associated ones, are listed in Additional file 2.

### Association of drug response with breast cancer subtype and ER status

All drugs were tested for association with breast cancer cell line subtypes. First, cell lines were grouped into luminal, basal, ERBB2-overexpressing and normal-like on the basis of intrinsic subtypes as previously reported (Hollestelle et al. 2010b). Statistical testing was performed either in BRB Array Tools version 4.2.1 Class Comparison using a T test or in Analyse-it version 2.26 (Leeds, UK) for Chi Square tests. To test whether ER protein expression was significantly associated with drug response, a Mann–Whitney test was used for linear IC_50_ profiles and a Chi Square or Fisher’s Exact test for two-group IC_50_ profiles. Previously published ER protein expression data (Riaz et al. 2013) was used as a categorical variable for ER status.

## Additional files

10.1186/s40064-015-1406-8 Detailed results of the drug screening and additional information regarding the data analysis. This table contains all calculated IC_50_ values for each drug and cell line. Furthermore the plotted IC_50_ values per drug are displayed and the categorization of the cell lines into resistant and sensitive per drug for the pathway analysis are listed.
10.1186/s40064-015-1406-8 Pathways associated with drugs. This file lists all Biocarta and KEGG pathways that are significantly associated with at least one drug. Pathways that are more significantly associated with a breast cancer subtype are in italic. The p-values given are for the drug-pathway association.

## Abbreviations

ABCA1: ATP-binding cassette, sub-family A (ABC1), member 1
c-Met: MET proto-oncogene
DMSO: dimethyl sulfoxide
DNA: deoxyribonucleic acid
EGFR: epidermal growth factor receptor
ER: estrogen receptor
ERBB2: Erb-b2 receptor tyrosine kinase 2
ERR1: estrogen-related receptor alpha
FBS: fetal bovine serum
FGFR: fibroblast growth factor receptor
HDAC: histone deacetylase
HER2: human epidermal growth factor receptor 2, Erb-b2 receptor tyrosine kinase 2
Her2-/neu: Erb-b2 receptor tyrosine kinase 2
IC_50_: inhibitory concentration 50, half maximal inhibitory concentration
KEGG: Kyoto Encyclopedia of Genes and Genomes
MDM2: MDM2 proto-oncogene, E3 ubiquitin protein ligase
NaCl: sodium chloride
NaOH: sodium hydroxide
NCBI: National Center for Biotechnology Information
PARP: poly ADP ribose polymerase
PR: progesterone receptor
RET: ret proto-oncogene
RNA: ribonucleic acid
RPMI: Roswell Park Memorial Institute
SRB: sulforhodamine B
TCA: trichloroacetic acid
TP53: tumor protein p53
TRIS: tris(hydroxymethyl)aminomethane
VEGFR: vascular endothelial growth factor receptor, kinase insert domain receptor
VEGFR2: vascular endothelial growth factor receptor 2, kinase insert domain receptor

## References

[CR1] Al-Hajj M, Wicha MS, Benito-Hernandez A, Morrison SJ, Clarke MF (2003). Prospective identification of tumorigenic breast cancer cells. Proc Natl Acad Sci.

[CR2] Armand J-P, Burnett AK, Drach J, Harousseau J-L, Löwenberg B, San Miguel J (2007). The emerging role of targeted therapy for hematologic malignancies: update on bortezomib and tipifarnib. Oncologist.

[CR3] Bast RC, Ravdin P, Hayes DF, Bates S, Fritsche H, Jessup JM, Kemeny N, Locker GY, Mennel RG, Somerfield MR (2001). 2000 update of recommendations for the use of tumor markers in breast and colorectal cancer: clinical practice guidelines of the American Society of Clinical Oncology. J Clin Oncol.

[CR4] Becker JP, Weiss J, Theile D (2014). Cisplatin, oxaliplatin, and carboplatin unequally inhibit in vitro mRNA translation. Toxicol Lett.

[CR5] Berry DA, Cronin KA, Plevritis SK, Fryback DG, Clarke L, Zelen M, Mandelblatt JS, Yakovlev AY, Habbema JDF, Feuer EJ (2005). Effect of screening and adjuvant therapy on mortality from breast cancer. N Engl J Med.

[CR6] Christman JK (2002). 5-Azacytidine and 5-aza-2′-deoxycytidine as inhibitors of DNA methylation: mechanistic studies and their implications for cancer therapy. Oncogene.

[CR7] Cohen RB (2003). Epidermal growth factor receptor as a therapeutic target in colorectal cancer. Clin Colorectal Cancer.

[CR8] Creusot F, Acs G, Christman JK (1982). Inhibition of DNA methyltransferase and induction of Friend erythroleukemia cell differentiation by 5-azacytidine and 5-aza-2′-deoxycytidine. J Biol Chem.

[CR9] Desmedt C, Voet T, Sotiriou C, Campbell PJ (2012). Next-generation sequencing in breast cancer: first take home messages. Curr Opin Oncol.

[CR10] Fornari FA, Randolph JK, Yalowich JC, Ritke MK, Gewirtz DA (1994). Interference by doxorubicin with DNA unwinding in MCF-7 breast tumor cells. Mol Pharmacol.

[CR11] Frederick CA, Williams LD, Ughetto G, van der Marel GA, van Boom JH, Rich A, Wang AH (1990). Structural comparison of anticancer drug-DNA complexes: adriamycin and daunomycin. Biochemistry (Mosc).

[CR12] Glendenning J, Tutt A (2011). PARP inhibitors–current status and the walk towards early breast cancer. Breast Edinb Scotl.

[CR13] Goeman JJ, van de Geer SA, de Kort F, van Houwelingen HC (2004). A global test for groups of genes: testing association with a clinical outcome. Bioinformatics.

[CR14] Gonzalez-Angulo AM, Morales-Vasquez F, Hortobagyi GN (2007). Overview of resistance to systemic therapy in patients with breast cancer. Adv Exp Med Biol.

[CR15] Gramza AW, Balasubramaniam S, Fojo AT, Ward J, Wells SA (2013) Phase I/II trial of vandetanib and bortezomib in adults with locally advanced or metastatic medullary thyroid cancer: Phase I results. J Clin Oncol 29:2011 (suppl; abstr 5565) 10.1634/theoncologist.2018-0452 PMC6324636 30297385

[CR16] Hagiwara H, Sunada Y (2004). Mechanism of taxane neurotoxicity. Breast Cancer.

[CR17] Hajihassan Z, Rabbani-Chadegani A (2009). Studies on the binding affinity of anticancer drug mitoxantrone to chromatin, DNA and histone proteins. J Biomed Sci.

[CR18] Heiser LM, Sadanandam A, Kuo WL, Benz SC, Goldstein TC, Ng S, Gibb WJ, Wang NJ, Ziyad S, Tong F, Bayani N, Hu Z, Billig JI, Dueregger A, Lewis S, Jakkula L, Korkola JE, Durinck S, Pepin F, Guan Y, Purdom E, Neuvial P, Bengtsson H, Wood KW, Smith PG, Vassilev LT, Hennessy BT, Greshock J, Bachman KE, Hardwicke MA (2012). Subtype and pathway specific responses to anticancer compounds in breast cancer. Proc Natl Acad Sci USA.

[CR19] Hollestelle A, Elstrodt F, Timmermans M, Sieuwerts AM, Klijn JGM, Foekens JA, den Bakker MA, Schutte M (2010). Four human breast cancer cell lines with biallelic inactivating α-catenin gene mutations. Breast Cancer Res Treat.

[CR20] Hollestelle A, Nagel JHA, Smid M, Lam S, Elstrodt F, Wasielewski M, Ng SS, French PJ, Peeters JK, Rozendaal MJ, Riaz M, Koopman DG, ten Hagen TLM, de Leeuw BHCGM, Zwarthoff EC, Teunisse A, van der Spek PJ, Klijn JGM, Dinjens WNM, Ethier SP, Clevers H, Jochemsen AG, den Bakker MA, Foekens JA, Martens JWM, Schutte M (2010). Distinct gene mutation profiles among luminal-type and basal-type breast cancer cell lines. Breast Cancer Res Treat.

[CR21] Huang Y, Rizzo RC (2012). A water-based mechanism of specificity and resistance for lapatinib with ErbB family kinases. Biochemistry (Mosc).

[CR22] Huynh H, Ngo VC, Fargnoli J, Ayers M, Soo KC, Koong HN, Thng CH, Ong HS, Chung A, Chow P, Pollock P, Byron S, Tran E (2008). Brivanib alaninate, a dual inhibitor of vascular endothelial growth factor receptor and fibroblast growth factor receptor tyrosine kinases, induces growth inhibition in mouse models of human hepatocellular carcinoma. Clin Cancer Res.

[CR23] Jansen MP, Foekens JA, van Staveren IL, Dirkzwager-Kiel MM, Ritstier K, Look MP, Meijer-van Gelder ME, Sieuwerts AM, Portengen H, Dorssers LC, Klijn JG, Berns EM (2005). Molecular classification of tamoxifen-resistant breast carcinomas by gene expression profiling. J Clin Oncol.

[CR24] Jones RJ, Gu D, Bjorklund CC, Kuiatse I, Remaley AT, Bashir T, Vreys V, Orlowski RZ (2013). The novel anticancer agent JNJ-26854165 induces cell death through inhibition of cholesterol transport and degradation of ABCA1. J Pharmacol Exp Ther.

[CR25] Kangaspeska S, Hultsch S, Edgren H, Nicorici D, Murumagi A, Kallioniemi O (2012). Reanalysis of RNA-sequencing data reveals several additional fusion genes with multiple isoforms. PLoS One.

[CR26] Katayama R, Aoyama A, Yamori T, Qi J, Oh-hara T, Song Y, Engelman JA, Fujita N (2013). Cytotoxic activity of tivantinib (ARQ 197) Is not due solely to c-MET inhibition. Cancer Res.

[CR27] Keyvanjah K, DePrimo SE, Harmon CS, Huang X, Kern KA, Carley W (2012). Soluble KIT correlates with clinical outcome in patients with metastatic breast cancer treated with sunitinib. J Transl Med.

[CR28] Kittaneh M, Montero AJ, Gluck S (2013). Molecular profiling for breast cancer: a comprehensive review. Biomark Cancer.

[CR29] Kumamoto K, Spillare EA, Fujita K, Horikawa I, Yamashita T, Appella E, Nagashima M, Takenoshita S, Yokota J, Harris CC (2008). Nutlin-3a activates p53 to both down-regulate inhibitor of growth 2 and up-regulate mir-34a, mir-34b, and mir-34c expression, and induce senescence. Cancer Res.

[CR30] Lehman JA, Hauck PM, Gendron JM, Batuello CN, Eitel JA, Albig A, Kadakia MP, Mayo LD (2013). Serdemetan antagonizes the Mdm2-HIF1α axis leading to decreased levels of glycolytic enzymes. PLoS One.

[CR31] Lemoine M, Younes A (2010). Histone deacetylase inhibitors in the treatment of lymphoma. Discov Med.

[CR32] Lichtenberg FR (2009). The effect of new cancer drug approvals on the life expectancy of American cancer patients, 1978–2004. Econ Innov New Technol.

[CR33] Lichtenberg FR (2011). Despite steep costs, payments for new cancer drugs make economic sense. Nat Med.

[CR34] Longley DB, Harkin DP, Johnston PG (2003). 5-fluorouracil: mechanisms of action and clinical strategies. Nat Rev Cancer.

[CR35] Lyko F, Brown R (2005). DNA methyltransferase inhibitors and the development of epigenetic cancer therapies. J Natl Cancer Inst.

[CR36] Momparler RL, Karon M, Siegel SE, Avila F (1976). Effect of adriamycin on DNA, RNA, and protein synthesis in cell-free systems and intact cells. Cancer Res.

[CR37] Nelson MH, Dolder CR (2006). Lapatinib: a novel dual tyrosine kinase inhibitor with activity in solid tumors. Ann Pharmacother.

[CR38] Ogata H, Goto S, Sato K, Fujibuchi W, Bono H, Kanehisa M (1999). KEGG: Kyoto encyclopedia of genes and genomes. Nucleic Acids Res.

[CR39] Parker JS, Mullins M, Cheang MC, Leung S, Voduc D, Vickery T, Davies S, Fauron C, He X, Hu Z, Quackenbush JF, Stijleman IJ, Palazzo J, Marron JS, Nobel AB, Mardis E, Nielsen TO, Ellis MJ, Perou CM, Bernard PS (2009). Supervised risk predictor of breast cancer based on intrinsic subtypes. J Clin Oncol.

[CR40] Perilongo G, Maibach R, Shafford E, Brugieres L, Brock P, Morland B, de Camargo B, Zsiros J, Roebuck D, Zimmermann A, Aronson D, Childs M, Widing E, Laithier V, Plaschkes J, Pritchard J, Scopinaro M, MacKinlay G, Czauderna P (2009). Cisplatin versus cisplatin plus doxorubicin for standard-risk hepatoblastoma. N Engl J Med.

[CR41] Ponti D, Zaffaroni N, Capelli C, Daidone MG (2006). Breast cancer stem cells: an overview. Eur J Cancer (Oxford, England: 1990).

[CR42] Previati M, Manfrini M, Galasso M, Zerbinati C, Palatini J, Gasparini P, Volinia S (2013). Next generation analysis of breast cancer genomes for precision medicine. Cancer Lett.

[CR43] R_Core_Team (2013) R: A Language and Environment for Statistical Computing. Vienna, Austria. http://www.R-project.org/

[CR44] Radovich M, Clare SE, Atale R, Pardo I, Hancock BA, Solzak JP, Kassem N, Mathieson T, Storniolo AM, Rufenbarger C, Lillemoe HA, Blosser RJ, Choi MR, Sauder CA, Doxey D, Henry JE, Hilligoss EE, Sakarya O, Hyland FC, Hickenbotham M, Zhu J, Glasscock J, Badve S, Ivan M, Liu Y, Sledge GW, Schneider BP (2013). Characterizing the heterogeneity of triple-negative breast cancers using microdissected normal ductal epithelium and RNA-sequencing. Breast Cancer Res Treat.

[CR45] Riaz M, van Jaarsveld MT, Hollestelle A, Prager-van der Smissen WJ, Heine AA, Boersma AW, Liu J, Helmijr J, Ozturk B, Smid M, Wiemer EA, Foekens JA, Martens JW (2013). MicroRNA expression profiling of 51 human breast cancer cell lines reveals subtype and driver mutation-specific miRNAs. Breast Cancer Res.

[CR46] Saldanha AJ (2004). Java Treeview-extensible visualization of microarray data. Bioinformatics.

[CR47] Sathornsumetee S, Rich JN (2006). Vandetanib, a novel multitargeted kinase inhibitor, in cancer therapy. Drugs Today Barc.

[CR48] Scagliotti GV, Novello S, von Pawel J (2013). The emerging role of MET/HGF inhibitors in oncology. Cancer Treat Rev.

[CR49] Shangary S, Wang S (2009). Small-molecule inhibitors of the MDM2-p53 protein-protein interaction to reactivate p53 function: a novel approach for cancer therapy. Annu Rev Pharmacol Toxicol.

[CR50] Sieuwerts AM, Kraan J, Bolt J, van der Spoel P, Elstrodt F, Schutte M, Martens JWM, Gratama J-W, Sleijfer S, Foekens JA (2009). Anti-epithelial cell adhesion molecule antibodies and the detection of circulating normal-like breast tumor cells. J Natl Cancer Inst.

[CR51] Sorlie T, Perou CM, Tibshirani R, Aas T, Geisler S, Johnsen H, Hastie T, Eisen MB, van de Rijn M, Jeffrey SS, Thorsen T, Quist H, Matese JC, Brown PO, Botstein D, Lonning PE, Borresen-Dale AL (2001). Gene expression patterns of breast carcinomas distinguish tumor subclasses with clinical implications. Proc Natl Acad Sci USA.

[CR52] Stordal B, Davey M (2007). Understanding cisplatin resistance using cellular models. IUBMB Life.

[CR53] Stordal B, Pavlakis N, Davey R (2007). A systematic review of platinum and taxane resistance from bench to clinic: an inverse relationship. Cancer Treat Rev.

[CR54] Teicher BA, Ara G, Herbst R, Palombella VJ, Adams J (1999). The proteasome inhibitor PS-341 in cancer therapy. Clin Cancer Res Off J Am Assoc Cancer Res.

[CR55] The Cancer Genome Atlas Network (2012). Comprehensive molecular portraits of human breast tumours. Nature.

[CR56] Van’t Veer LJ, Dai H, van de Vijver MJ, He YD, Hart AA, Mao M, Peterse HL, van der Kooy K, Marton MJ, Witteveen AT, Schreiber GJ, Kerkhoven RM, Roberts C, Linsley PS, Bernards R, Friend SH (2002). Gene expression profiling predicts clinical outcome of breast cancer. Nature.

[CR57] Voigt W (2005). Sulforhodamine B assay and chemosensitivity. Methods Mol Med.

[CR58] Wang Y, Klijn JGM, Zhang Y, Sieuwerts AM, Look MP, Yang F, Talantov D, Timmermans M, Meijer-van Gelder ME, Yu J, Jatkoe T, Berns EMJJ, Atkins D, Foekens JA (2005). Gene-expression profiles to predict distant metastasis of lymph-node-negative primary breast cancer. Lancet Lond Engl.

